# Stereotactic body radiotherapy of bone metastases in patients with head and neck squamous cell carcinoma – A multicenter cohort study

**DOI:** 10.1016/j.ctro.2026.101336

**Published:** 2026-10-30

**Authors:** Alexander Rühle, Sebastian Schäfer, Isabell Prager, Mauro Loi, Pierluigi Bonomo, Viola Salvestrini, Marek Slavik, Petr Burkon, Fabian Lohaus, Kenneth Klischies, Olaf Wittenstein, Anna Sabrina Schunn, Sophia Drabke, Thomas Weissmann, Jochen Willner, Camilla von Wachter, Esmée L. Looman, Panagiotis Balermpas, Matthias Guckenberger, Sebastian Michael Ritter, Christos Moustakis, Ahmed Allam Mohamed, Thomas B. Brunner, Oliver Blanck, Judit Boda-Heggemann, Nils H. Nicolay, Franziska Nägler

**Affiliations:** a Department of Radiation Oncology, University Medical Center Leipzig, Leipzig, Germany; b Comprehensive Cancer Center Central Germany, Partner Site Leipzig, Leipzig, Germany; c Radiation Oncology Unit, Azienda Ospedaliero-Universitaria Careggi, University of Florence, Florence, Italy; d Department of Radiation Oncology, Masaryk Memorial Cancer Institute and Medical Faculty, Masaryk University, Brno, Czech Republic; e Department of Radiotherapy and Radiation Oncology, Faculty of Medicine and University Hospital Carl Gustav Carus, TUD Dresden University of Technology, Dresden, Germany; f National Center for Tumor Diseases (NCT), NCT/UCC Dresden, a partnership between DKFZ, Faculty of Medicine and University Hospital Carl Gustav Carus, TUD Dresden University of Technology, and Helmholtz-Zentrum Dresden-Rossendorf (HZDR), Germany; g OncoRay – National Center for Radiation Research in Oncology, Faculty of Medicine and University Hospital Carl Gustav Carus, TUD Dresden University of Technology, Helmholtz-Zentrum Dresden-Rossendorf, Dresden, Germany; h German Cancer Consortium (DKTK), Partner Site Dresden, and German Cancer Research Center (DKFZ), Heidelberg, Germany; i Department of Radiation Oncology, University Medical Center Schleswig-Holstein, Kiel, Germany; j Department of Radiotherapy and Radiation Oncology, University Medical Center Mainz, Mainz, Germany; k Department of Radiation Oncology, Universitätsklinikum Erlangen, Friedrich-Alexander-Universität Erlangen-Nürnberg, Erlangen, Germany; l Department of Radiotherapy, Klinikum Bayreuth GmbH, Bayreuth, Germany; m Department of Radiation Oncology, University Hospital Zurich, University of Zurich, Zurich, Switzerland; n Department of Radiotherapy and Radio-Oncology, University Medical Center Augsburg, Augsburg, Germany; o Department of Radiation Oncology, RWTH Aachen University Hospital, Aachen, Germany; p Department of Therapeutic Radiology and Oncology, Comprehensive Cancer Center, Medical University of Graz, Graz, Austria; q Department of Radiation Oncology, University Medicine Mannheim, Medical Faculty Mannheim, Heidelberg University, Mannheim, Germany; r DKFZ Hector Cancer Institute at the University Medical Center Mannheim, Mannheim, Germany

**Keywords:** Bone metastases, Stereotactic body radiation treatment, SBRT, Metastasis-directed therapy, MDT, Head and neck squamous cell carcinoma, HNSCC

## Abstract

**Purpose:**

Bone metastases from head and neck squamous cell carcinoma (HNSCC) are a rare clinical entity, with limited data guiding management. This study aimed to evaluate the oncological outcomes of stereotactic body radiotherapy (SBRT) for this rare condition.

**Methods:**

Patients who received SBRT for HNSCC-derived bone metastases between 2015 and 2024 were analyzed. Overall survival (OS) and progression-free survival (PFS) were estimated using the Kaplan-Meier method. The cumulative incidence of local failures (LF) and post-SBRT fractures was analyzed with death as competing event. Adverse events were graded according to CTCAE v5.0.

**Results:**

Thirty-four patients with 54 irradiated bone metastases from 11 European centers were included. Median age was 63 years (range, 39–86); and the most common primary sites were the oropharynx (*n* = 12, 35.3%), nasopharynx (*n* = 8, 23.5%), and oral cavity (*n* = 5, 14.7%). The median prescribed biologically effective dose (BED_10_) was 60.0 Gy (IQR, 56.0–72.0 Gy). After a median follow-up of 23.3 months in patients alive at last contact, 2-year OS and PFS were 41.2% (95% CI, 24.3–57.3%) and 8.8% (95% CI, 2.3–21.1%), while the 2-year cumulative incidence of LF and fractures was 14.1% (95% CI, 3.0–27.8%) and 1.9% (95% CI, 0.0–6.9%). In exploratory analyses, patients treated for >1 bone metastases had inferior OS compared to those with a single treated bone metastasis (HR 2.58; 95% CI, 1.08–6.15; *p* = 0.033). Grade 3 adverse events occurred in one patient (2.9%), with no grade 4–5 events.

**Conclusion:**

Although PFS remained limited, SBRT was associated with high local control and a low observed rate of severe adverse events in patients with HNSCC-derived bone metastases. Prospective studies are warranted to validate these findings and optimize patient selection.

## Highlights

• Multicenter cohort of 34 patients with 54 SBRT-treated HNSCC bone metastases.
• 2-year OS and PFS were 41.2% (95% CI, 24.3–57.3%) and 8.8% (95% CI, 2.3–21.1%).
• 2-year incidence of local failures was 14.1% (95% CI, 3.0–27.8%).
• Grade 3 adverse events occurred in one patient (2.9%), with no grade 4–5 events.
• In an exploratory analysis, treatment of >1 bone metastases was associated with shorter OS.

## 1 Introduction

Head and neck squamous cell carcinoma (HNSCC) is characterized by aggressive locoregional behavior and a substantial risk of disease recurrence [1]. While locoregional failure remains the dominant pattern of relapse, distant metastases are increasingly recognized as a competing first event, particularly in patients with advanced primary tumors and extensive nodal disease [2], [3]. Approximately 15–20% of patients with locoregionally advanced HNSCC develop distant metastases as the initial site of progression, and up to half of these patients present with a limited metastatic burden consistent with an oligometastatic disease state [2], [4], [5]. This intermediate clinical phenotype has attracted growing interest, as it may offer a window for metastasis-directed treatment (MDT) in carefully selected patients [6].

Stereotactic body radiotherapy (SBRT) has emerged as a key MDT in oligometastatic cancer due to its ability to deliver high radiation doses with steep dose gradients and limited side effects. In oligometastatic HNSCC, available evidence suggests that SBRT can achieve durable local control with acceptable safety, particularly for pulmonary metastases [7]. However, most published series and the only randomized phase II trial in this setting predominantly included patients with lung-only metastatic disease, limiting the generalizability of these findings to other metastatic sites [2], [7], [8], [9], [10].

Compared to lung metastases, bone metastases occur less frequently in HNSCC but are clinically relevant because of their association with pain, risk of skeletal-related events, and potential impairment of quality of life [11], [12], [13]. A recent Head and Neck Cancer International Group (HNCIG) survey found that SBRT is the preferred treatment technique for bone metastases in oligometastatic head and neck cancer [14]. However, the available evidence on SBRT for HNSCC-derived bone metastases is largely derived from cohorts of patients with nasopharyngeal carcinoma, whereas data for non-nasopharyngeal cancer entities are limited and often originate from heterogeneous cohorts that also include SBRT for metastases at other sites and from different primary cancers [15], [16], [17], [18].

Given the limited evidence on the efficacy and tolerability of SBRT for HNSCC-derived bone metastases, we conducted a multicenter retrospective cohort study to evaluate the oncological outcomes and safety of SBRT in this setting and thereby address an important evidence gap to inform multidisciplinary clinical decision-making.

## 2 Material and methods

### 2.1 Study design

This multicenter cohort study was initiated by the working group for radiosurgery and stereotactic radiotherapy of the German Society for Radiation Oncology (DEGRO). Patients with HNSCC of the oral cavity, nasopharynx, oropharynx, hypopharynx, paranasal sinus, or with unknown primary, who were treated with SBRT for at least one bone metastasis between 2015 and 2024 were retrospectively analyzed. The study was performed at 11 institutions in Germany, Austria, Switzerland, Italy, and the Czech Republic, and it followed the STROBE guidelines for reporting observational studies (**Supplementary Table 1**). Ethical approval was obtained from the respective institutional ethics committee at each participating center, with central approval by the Ethics Advisory Board at Leipzig University (reference number: 127/24-ek).

Diagnosis of bone metastasis was determined by positron emission tomography (PET/CT), magnetic resonance imaging (MRI), or computed tomography (CT), provided that the multidisciplinary tumor board considered the lesion most likely to be HNSCC-derived. The classification of oligometastatic disease was based on the ESTRO/EORTC consensus criteria [19]. The previous treatment of the underlying HNSCC, systemic therapies during SBRT and thereafter, and occurrence of further metastases were recorded. Comprehensive SBRT parameters were documented in accordance with the International Commission on Radiation Units and Measurements (ICRU) report 91 [20], [21]. For the target volume, a minimum prescription biologically effective dose (BED_10_, α/β = 10 Gy) of 48 Gy in a maximum of 12 fractions was required, thereby excluding palliative concepts. BED_10_ was calculated with the formula BED = n × d × (1 + d/(α/β)) with n = number of fractions, and d = dose per fraction.

### 2.2 Response assessment

The clinical data and radiological findings from routine examinations performed after SBRT (including CT, MRI, and PET scans) were retrospectively evaluated regarding local and distant tumor progression and the occurrence of fractures. Local failure (LF) was defined as in-field progression in radiological imaging (CT, MRI, or PET-CT); bone metastases without progression were censored at the time of most recent imaging independently for each lesion. Progression-free survival (PFS) was defined as the time from completion of SBRT to imaging-confirmed tumor progression or death from any cause, with patients without documented progression being censored at the date of their last imaging follow-up. Overall survival (OS) was calculated from the completion of SBRT to death; patients alive at last contact were censored at the recorded last-known-alive date. Due to the retrospective multicenter design, imaging was assessed locally as part of routine follow-up. There was no central imaging review for the present analysis, and standardized response criteria (e.g., SPINO) were not applied uniformly. Equivocal post-SBRT imaging changes were assessed by the treating centers based on the local radiology reports and, where applicable, multidisciplinary tumor board decisions. Treatment-related adverse events were graded according to the Common Terminology Criteria for Adverse Events (CTCAE) v5.0 and were classified as acute if they occurred within the first 90 days following the initiation of SBRT, and as chronic if they developed thereafter. Adverse events were reported both per patient (highest grade per patient) and per treated lesion.

### 2.3 Statistical analysis

All analyses were performed in Python (version 3.12.7) using custom scripts. Data handling and preprocessing were implemented with pandas (v2.2.3) and NumPy (v2.2.1). Time-to-event analyses were conducted with lifelines (v0.30.0), and figures were generated using Matplotlib (v3.10.0). Continuous variables were summarized as median with inter-quartile range (IQR). OS and PFS were estimated using the Kaplan-Meier method, and survival estimates at 24 months were reported with 95% confidence intervals (95% CI). Follow-up time was calculated using the reverse Kaplan-Meier method and additionally reported as the median follow-up of patients alive at last contact. The cumulative incidence of LF and post-SBRT fractures was analyzed in a competing-risks framework, with death as a competing event. Cumulative incidence functions were estimated using the Aalen-Johansen estimator, and 24-month incidence estimates were reported with 95% CI derived from nonparametric bootstrapping at the patient level (2000 resamples of patients including all of their lesions) to account for multiple lesions per patient. As a sensitivity analysis, cumulative incidences were additionally estimated for the first treated lesion per patient. No imputation methods were applied for missing data.

The association of pre-specified clinical variables with OS was assessed by univariable Cox proportional hazards regression, with results reported as hazard ratios (HR) with 95% CI. The following variables were tested: age (continuous, per year), sex (male vs. female), Karnofsky performance status (dichotomized as ≤70% vs. ≥80%), primary tumor site (with oropharyngeal carcinoma as reference), time from initial diagnosis to bone metastasis (continuous, in months), synchronous vs. metachronous bone metastasis, single vs. multiple treated bone metastases, total metastatic burden (1 vs. 2–5 vs. >5), bone-only disease (yes vs. no) and concurrent immunotherapy (yes vs. no). Concurrent immunotherapy was defined as the administration of immune checkpoint inhibitors (ICI) within 4 weeks before or after the SBRT regimen. All statistical tests were two-sided, and a significance level of *p* < 0.05 was considered statistically significant. As these analyses were exploratory, no corrections for multiple testing were performed, and results should be interpreted as hypothesis-generating.

## 3 Results

### 3.1 Study cohort

A total of 34 patients with 54 treated lesions were included in the analysis. Patient and treatment characteristics are shown in Table 1. The median age at first SBRT was 63 years, and 31 patients (91.2%) were male. The most common primary sites were the oropharynx (*n* = 12, 35.3%), nasopharynx (*n* = 8, 23.5%), and oral cavity (*n* = 5, 14.7%). Among the 12 patients with oropharynx carcinoma, 5 (41.7%) had a p16-positive carcinoma. A median of 1 (IQR, 1–2) bone metastasis per patient was treated. In terms of total metastatic burden including non-bone metastatic sites, 16 patients (47.1%) had a single metastasis, 15 (44.1%) had 2–5 metastases, and 3 (8.8%) had >5 metastases. All known metastatic sites were treated with MDT in 32 patients (94.1%), including 10 of 12 patients with non-bone metastases. In the remaining 2 patients (5.9%), both with non-bone metastases, only selected lesions were treated. At least one line of systemic therapy was documented in 28 patients (82.4%), and 19 patients (55.9%) received ICI during the course of their disease, in 14 patients as a subsequent line after SBRT. Relative to the index SBRT course, a line of systemic therapy was started before SBRT in the metastatic setting in 6 patients, within ±4 weeks of SBRT in 4 patients, and after SBRT in 22 patients; in 2 patients, the exact start date was not documented. Including ongoing treatment, 18 patients (52.9%) received systemic therapy within ±4 weeks of SBRT, 10 of them with ICI. According to the ESTRO/EORTC classification [19], 15 patients (44.1%) presented with synchronous oligometastatic disease, 11 (32.4%) with (repeat) oligorecurrence, and 8 (23.5%) with (repeat) oligoprogression.

**Table 1:** Patient and treatment characteristics.

Variable	Value
**Patients, n**	34 (100%)
**Age, median (range), years**	63 (39–86)
**Sex**	
Male	31 (91.2%)
Female	3 (8.8%)
**KPS, median (IQR), % (*n* = 31)**	90 (80–90)
**PD-L1 expression (CPS)**	
0	5 (14.7%)
1–19	9 (26.5%)
≥20	3 (8.8%)
Unknown	17 (50.0%)
**HNSCC tumor site**	
Oropharynx	12 (35.3%)
Nasopharynx	8 (23.5%)
Oral cavity	5 (14.7%)
Cancer of unknown primary	3 (8.8%)
Hypopharynx	3 (8.8%)
Paranasal sinus	3 (8.8%)
**Treated BoM per patient**	
1	23 (67.6%)
2	4 (11.8%)
3	5 (14.7%)
4	2 (5.9%)
**Median treated BoM per patient (IQR)**	1 (1–2)
**Total number of metastases at the time of first SBRT**	
1	16 (47.1%)
2–5	15 (44.1%)
>5	3 (8.8%)
**Bone-only metastatic disease**	
Yes	22 (64.7%)
No	12 (35.3%)
**Systemic therapy within ± 4 weeks of SBRT**⁎	18 (52.9%)
Cytotoxic chemotherapy	12 (35.3%)
Immunotherapy	10 (29.4%)
No systemic therapy	16 (47.1%)
**Total treated BoM, n**	54 (100%)
**Time from initial diagnosis to BoM, median (IQR), months**	6.3 (1.5–11.4)
**Occurrence of BoM**	
Synchronous	20 (37.0%)
Metachronous	34 (63.0%)
**Type of metachronous presentation (*n* = 34)**	
Oligoprogression	6 (17.6%)
Oligorecurrence	21 (61.8%)
Repeat oligoprogression	5 (14.7%)
Repeat oligorecurrence	2 (5.9%)
Induced oligoprogression	0 (0.0%)
Induced oligorecurrence	0 (0.0%)
**BoM localization**	
Spine	34 (63.0%)
Pelvis	10 (18.5%)
Ribs	5 (9.3%)
Long bones	3 (5.6%)
Sternum	2 (3.7%)
**Characteristics of BoM**	
Radiological osteolytic	37 (68.5%)
Radiological osteoblastic	8 (14.8%)
Mixed osteolytic/osteoblastic	1 (1.9%)
Unknown	8 (14.8%)
**Soft tissue infiltration**	
Yes	5 (9.3%)
No	44 (81.5%)
Unknown	5 (9.3%)
**Spinal canal infiltration of spine metastases (n = 34)**	
Yes	1 (2.9%)
No	28 (82.4%)
Unknown	5 (14.7%)
**Symptomatic before SBRT**	
Yes	13 (24.1%)
No	32 (59.3%)
Unknown	9 (16.7%)
**Imaging before treatment planning**⁎	
Diagnostic CT	5 (9.3%)
MRI	37 (68.5%)
PET/CT	47 (87.0%)
**Treatment technique**	
C-arm based system (FF)	13 (24.1%)
C-arm based system (FFF)	36 (66.7%)
Robotic-based system	5 (9.3%)

Abbreviations: BoM, bone metastases; CPS, combined positive score; CT, computed tomography; FF(F), flattening filter (−free); HNSCC, head and neck squamous cell carcinoma; IQR, inter-quartile range; MRI, magnetic resonance imaging; PET, positron emission tomography; SBRT, stereotactic body radiotherapy.

⁎ Multiple responses possible.

Bone metastases predominantly involved the spine (34/54, 63.0%), followed by the pelvis (10/54, 18.5%) and ribs (5/54, 9.3%). Radiologically, most bone metastases were osteolytic (68.5%), whereas osteoblastic (14.8%) and mixed osteolytic/osteoblastic lesions (1.9%) were uncommon. Soft tissue infiltration was present in 5 of 54 treated bone metastases (9.3%), while spinal canal infiltration was observed in only one of 34 spinal metastases (2.9%). At the time of SBRT, 13 lesions (24.1%) were symptomatic. The median interval from initial HNSCC diagnosis to the development of bone metastases was 6.3 months (IQR, 1.5–11.4); 20 lesions (37.0%) were synchronous and 34 (63.0%) were metachronous. Pre-treatment imaging most commonly included PET/CT (87.0%) and MRI (68.5%).

The median GTV and PTV were 10.9 mL (IQR, 6.2–32.0) and 28.6 mL (IQR, 15.4–62.3). Most lesions were treated with 5-fraction SBRT regimens, with 40 Gy in 5 fractions (25.9%) and 35 Gy in 5 fractions (16.7%) representing the most commonly prescribed schedules. Less frequently used regimens included 48.5 Gy in 10 fractions (14.8%), 30 Gy in 3 fractions (13.0%), and 30 Gy in 5 fractions (13.0%). The median prescribed BED_10_ was 60.0 Gy (IQR, 56.0–72.0 Gy), while the median PTV D98%, D50%, and D2% were 33.1 Gy (IQR, 25.9–38.4 Gy), 36.3 Gy (IQR, 30.7–40.9 Gy), and 39.3 Gy (IQR, 33.0–42.1 Gy), respectively. Details of the dose parameters according to ICRU91 are presented in the **Supplementary Table 2**.

### 3.2 Oncological outcomes and adverse events

After a median follow-up of 51.2 months (95% CI, 23.7–102.1) using the reverse Kaplan-Meier method and of 23.3 months (range, 2.3–102.1) in the patients alive at last contact, 26 deaths, 28 disease progressions, and 7 LF were observed. The median OS and PFS were 18.0 and 3.0 months, respectively. Two-year OS and PFS were 41.2% (95% CI, 24.3–57.3%) and 8.8% (95% CI, 2.3–21.1%), respectively (Fig. 1). The 2-year cumulative incidence of LF was 14.1% (95% CI, 3.0–27.8%) (Fig. 2), and the prescribed BED_10_ of the 7 lesions with LF were 72.0 Gy (3×), 59.5 Gy, 56.0 Gy (2×), and 48.0 Gy. Four of them were localized in the spine, and 3 in the pelvis. At 24 months, only 7 lesions remained at risk, indicating that the 2-year estimates should be interpreted with caution. Restricted to the first treated lesion per patient, the 2-year cumulative incidence of LF was 12.7% (95% CI, 2.9–25.8%). Only one of 54 treated lesions (1.9%) developed a fracture at 4.6 months after SBRT, translating into a 2-year incidence of bone fractures of 1.9% (95% CI, 0.0–6.9%). The patient was treated for an osteolytic lesion in the spine, with 24 Gy in 2 fractions, prescribed to the 70% isodose, resulting in a BED_10_ of 52.8 Gy related to the prescription dose.

**Fig. 1:**
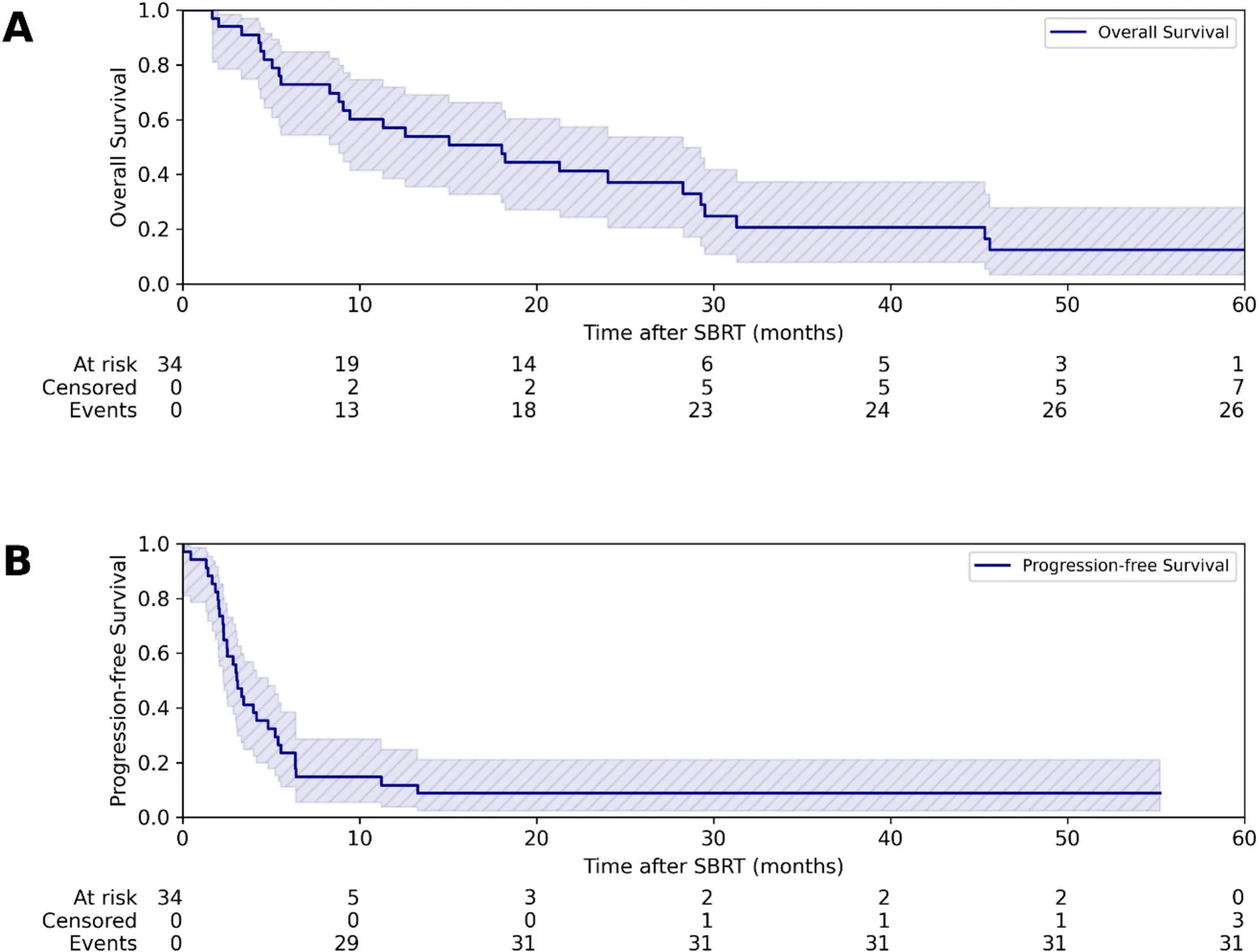
[A] Overall survival (OS) and [B] progression-free survival (PFS) for SBRT of bone metastases from HNSCC.

**Fig. 2:**
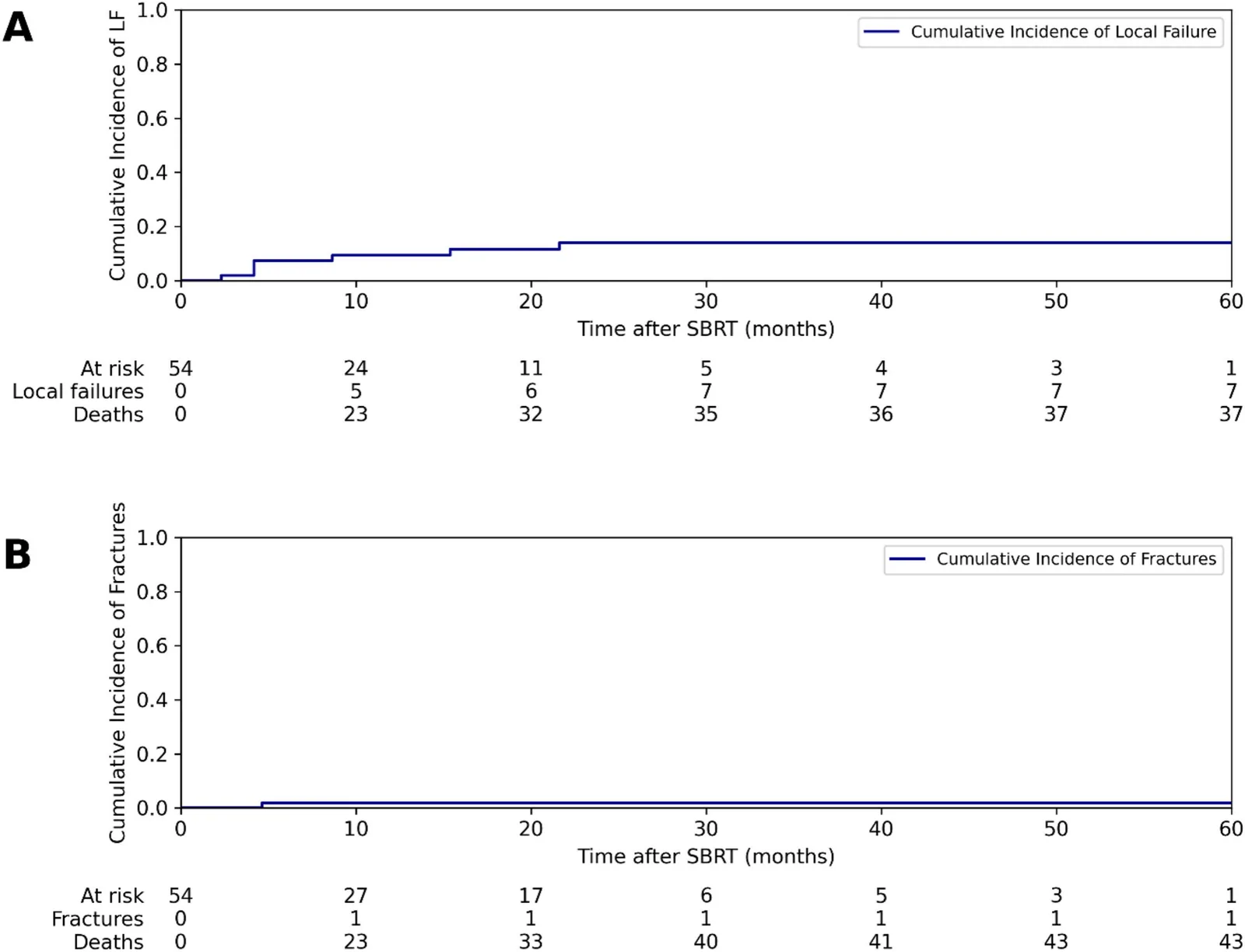
Cumulative incidence of [A] local failures and [B] fractures for SBRT of bone metastases from HNSCC, with death as competing event. Numbers at risk and deaths refer to treated lesions.

The results of the univariable Cox proportional hazards regression analysis are presented in Table 2. A Karnofsky performance status ≤70% (HR 3.70 [95% CI 1.31–10.46], *p* = 0.013) and the presence of multiple treated bone metastases (HR 2.58 [95% CI 1.08–6.15], *p* = 0.033) were both significantly associated with inferior OS in univariable analysis. Age, sex, primary tumor site, time from initial diagnosis to bone metastasis, synchronous versus metachronous bone metastases, total metastatic burden, bone-only disease, and concurrent immunotherapy were not significantly associated with OS. This also applied to total metastatic burden dichotomized as 1 vs. >1 metastases (HR 1.59 [95% CI 0.72–3.53], *p* = 0.247).

**Table 2:** **Univariable analyses for overall survival (OS).** A total of 34 patients were analyzed in the Cox proportional hazards regression analysis. There were 26 events. Karnofsky performance status was available for 31 patients (23 events).

Variable	HR	95% CI	*p*-value
**Age [years]**	1.03	0.99–1.07	0.165
**Karnofsky performance status**			
≥80%	Reference		
≤70%	3.70	1.31–10.46	**0.013**
**Time from initial diagnosis to BoM [months]**	0.99	0.98–1.01	0.315
**Sex**			
Male	Reference		
Female	1.56	0.46–5.35	0.476
**Primary tumor site**			
Oropharynx	Reference		
Nasopharynx	0.62	0.21–1.77	0.370
Oral cavity	1.36	0.36–5.13	0.649
Cancer of unknown primary	0.91	0.24–3.44	0.892
Hypopharynx	0.79	0.21–2.97	0.733
Paranasal sinus	1.15	0.24–5.40	0.863
**BoM occurrence**			
Synchronous	Reference		
Metachronous	1.02	0.47–2.22	0.962
**Number of treated BoM**			
1	Reference		
≥2	2.58	1.08–6.15	**0.033**
**Total metastatic burden**			
1	Reference		
2–5	1.45	0.62–3.38	0.392
>5	2.49	0.68–9.13	0.168
**Bone-only disease**			
Yes	Reference		
No	1.18	0.51–2.74	0.703
**Concurrent immunotherapy**			
Yes	Reference		
No	1.71	0.70–4.16	0.236

Abbreviations: BoM, bone metastasis; CI, confidence interval; HR, hazard ratio.

In an exploratory analysis, OS was significantly longer in patients with a single treated bone metastasis (2-year OS: 50.0% [95% CI, 28.2–68.4%] vs. 21.2% [95% CI, 3.3–49.3%]; *p* = 0.027 (log-rank test); Fig. 3). However, PFS did not differ significantly between the two groups (2-year PFS: 13.0% [95% CI, 3.3–29.7%] vs. 0.0% [CI not estimable]; HR 1.55 [95% CI, 0.74–3.27]; *p* = 0.235). Exploratory analyses stratified by primary tumor site (p16-positive oropharyngeal cancer vs. nasopharyngeal cancer vs. other sites) and concurrent ICI use are presented in the **Supplementary Data**; neither analysis demonstrated significant differences in outcomes.

**Fig. 3:**
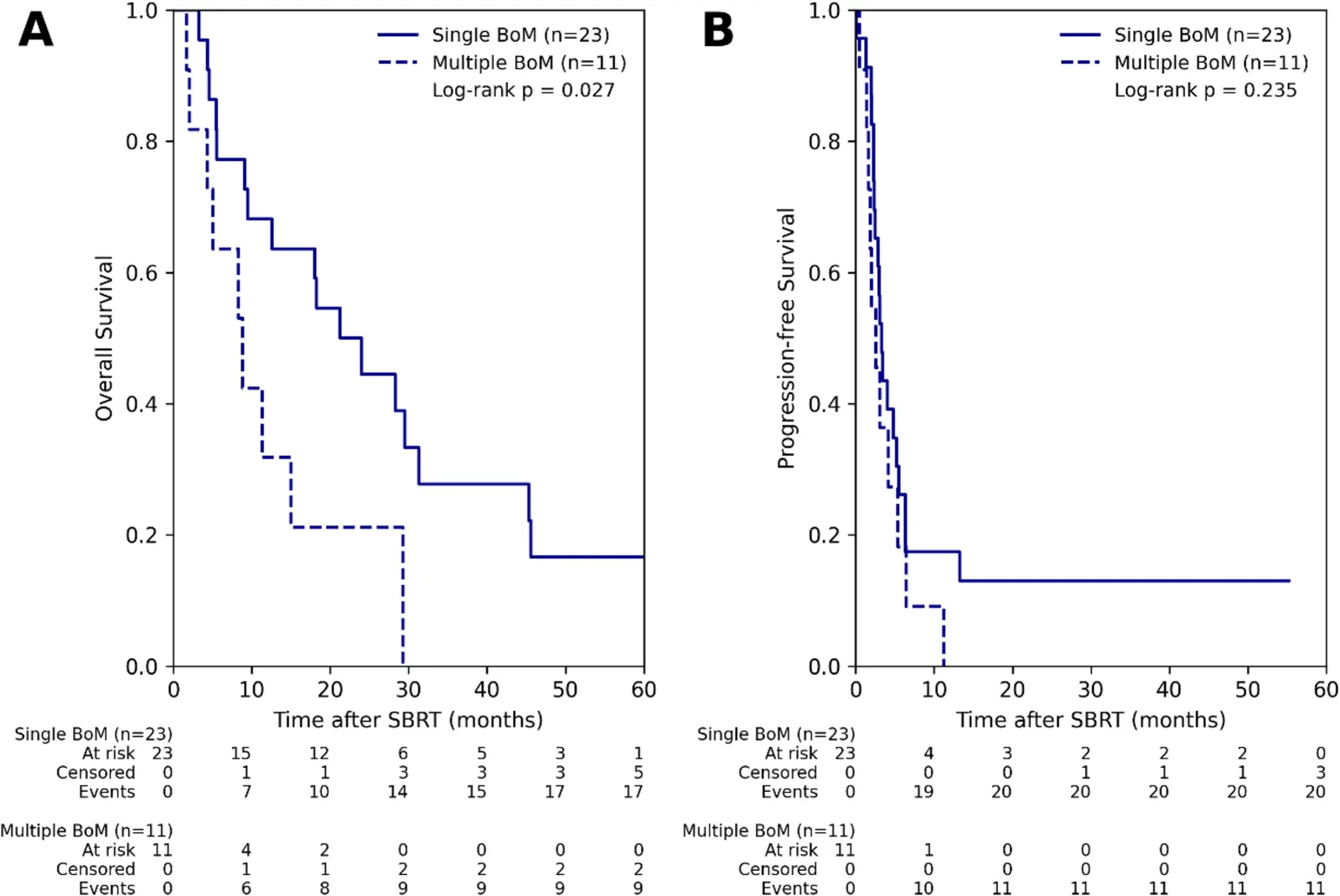
[A] Overall survival and [B] progression-free survival stratified by number of treated lesions for SBRT of bone metastases (BoM) from HNSCC.

Overall, treatment was well tolerated. Acute grade 1–2 adverse events were uncommon and consisted of grade 2 acute pain (3 patients, 8.8%) and mucositis (1 patient, 2.9%), as well as grade 1 diarrhea (1 patient, 2.9%) (Table 3). One patient (2.9%) experienced a grade 3 acute pain event, which occurred on the day of the last administered SBRT fraction. Accordingly, the crude incidence of acute grade 3 adverse events was 2.9% per patient and 1.9% per treated lesion, with no grade 4–5 adverse events observed.

**Table 3:** **SBRT-related acute and chronic adverse events.** Reported according to Common Terminology Criteria for Adverse Events (CTCAE) version 5.0. Adverse events were recorded for each treated lesion and are given per treated lesion (*n* = 54) / per patient (*n* = 34, highest grade per patient).

Adverse event	Grade 1 n (%)	Grade 2 n (%)	Grade 3 n (%)	Grade 4 n (%)	Grade 5 n (%)
Acute pain	–	7 (13.0%) / 3 (8.8%)	1 (1.9%) / 1 (2.9%)	–	–
Mucositis	–	2 (3.7%) / 1 (2.9%)	–	–	–
Diarrhea	1 (1.9%) / 1 (2.9%)	–	–	–	–
Late pathological fracture	1 (1.9%) / 1 (2.9%)	–	–	–	–

## 4 Discussion

In this multicenter cohort study, we evaluated oncological outcomes and safety of SBRT for bone metastases in HNSCC. Despite the rarity of this clinical scenario, our analysis represents one of the largest dedicated datasets focusing specifically on bone-directed SBRT in HNSCC. We found that SBRT was associated with high local control and low rates of severe adverse events and skeletal complications, while PFS remained limited, reflecting the systemic nature of the disease. In exploratory analyses, a single treated bone metastasis was associated with longer OS. As total metastatic burden was not associated with OS, this finding is likely influenced by patient selection and should be interpreted with caution.

Local control after SBRT of bone metastases in our cohort was favorable, with a 2-year cumulative incidence of LF of 14.1%. This is in line with the broader SBRT literature across solid tumors, where durable local control exceeding 80% at 2 years is frequently reported when ablative doses are applied [22], [23]. The capacity of SBRT to deliver high biologically effective doses with steep dose gradients appears particularly advantageous in bone lesions, where critical structures such as the spinal cord or major vessels often limit dose escalation with conventional fractionation. Among the 7 lesions with LF, the median prescribed BED_10_ was 59.5 Gy which is almost identical to the median prescribed BED_10_ of 60.0 Gy. Whether these LF primarily reflect adverse tumor biology, technical factors such as target delineation or treatment delivery, or other factors cannot be determined from the present study and warrants further investigation.

When placing our results into the context of nasopharynx cancer-dominated SBRT series, similarities and differences emerge. Published cohorts in metastatic nasopharynx cancer have demonstrated excellent local control rates and, in some reports, prolonged survival, likely reflecting both a more radiosensitive tumor biology and the availability of effective systemic therapy options [16], [24], [25]. However, the transferability of these findings to non-nasopharyngeal HNSCC has remained uncertain. Our data suggest that local efficacy of SBRT is comparably high even in non-nasopharyngeal HNSCC, whereas survival outcomes appear less favorable.

OS in our cohort was moderate, with a 2-year OS of 41.2%, which appears somewhat lower than that reported in SBRT cohorts of oligometastatic HNSCC with predominantly lung metastases [7], [8]. Pulmonary metastases are frequently associated with a more indolent clinical course and may represent a biologically distinct subgroup with more favorable tumor kinetics. In contrast, bone metastases have traditionally been regarded as a negative prognostic factor across many solid malignancies, including head and neck cancers [11], [26]. More recently, Xu et al. reported encouraging outcomes in a cohort of 94 patients with metastatic HNSCC treated with metastasis-directed radiotherapy in combination with PD-1–based immunotherapy and chemotherapy, demonstrating a 2-year OS of 70.1% [24]. However, the majority of patients had nasopharyngeal carcinoma (77.7%), nearly half presented with isolated lung metastases, and all patients received contemporary immunotherapy, limiting direct comparisons with the present study.

The safety profile of SBRT in our study was favorable, with very low rates of severe acute or late adverse events and a 2-year cumulative incidence of post-SBRT fractures of 1.9% (one fracture in 54 treated lesions). This is comparable to, or even lower than, fracture rates reported in broader spine SBRT series across tumor entities [22], [23]. Importantly, most lesions in our cohort were located in the spine, a region traditionally associated with elevated fracture risk following high-dose irradiation. The low fracture incidence observed here may reflect careful patient selection, adherence to dose constraints, and contemporary planning techniques.

Despite the limited PFS observed in our cohort, with a median PFS of 3.0 months and a 2-year PFS of 8.8%, prospective data continue to support the role of MDT in carefully selected patients with oligometastatic HNSCC. The randomized phase II GORTEC 2014–04 OMET trial enrolled patients with 1–3 oligometastases, the majority of whom had lung-only disease (82.6%), and compared SBRT alone with SBRT combined with EXTREME-based systemic therapy [8]. Median overall survival exceeded 40 months in both treatment arms, while median PFS reached 7.4 months with SBRT alone and 12.9 months with chemo-SBRT. Importantly, omission of upfront systemic therapy resulted in substantially lower rates of severe adverse events and quality-of-life deterioration without an apparent detriment in OS. These favorable outcomes likely reflect the highly selected nature of the study population, characterized predominantly by pulmonary oligometastases and limited metastatic burden. In contrast, our cohort consisted exclusively of patients with bone metastases, a disease manifestation traditionally associated with a less favorable prognosis.

More recently, So et al. reported a prospective phase II study combining SBRT with pembrolizumab in patients with PD-1–refractory recurrent or metastatic HNSCC. Notably, PFS was markedly superior when all known oligometastatic or oligoprogressive lesions were treated (median PFS 5.7 months) compared with irradiation of a single lesion only (median PFS 1.8 months), supporting a comprehensive MDT strategy rather than reliance on a potential abscopal effect [27]. Furthermore, patients treated to more than 50% of visible metastatic lesions achieved a median OS of 21.3 months, compared with 5.5 months in those with less extensive lesion coverage. These findings provide additional biological and clinical rationale for the ongoing PROLoNg trial, which evaluates the addition of SBRT to pembrolizumab in patients with oligometastatic HNSCC. The protocol recommends an ablative dose of 35 Gy in 5 fractions, while 24 Gy in 2 fractions and 20 Gy in a single fraction are permitted alternatives. As SBRT was predominantly used with ablative intent in our cohort, as indicated by the BED_10_ threshold of ≥48 Gy, the low proportion of symptomatic lesions (24.1%) and the treatment of all known metastatic sites in 32 of 34 patients, trials of palliative or prophylactic radiotherapy apply only to a limited extent. Nevertheless, a single fraction of 8 Gy remains a reasonable option for patients with predominantly palliative or prophylactic indications and limited life expectancy [28], [29], while 24 Gy in 2 fractions is a well-established alternative for selected ablative spine treatments [30]. The frequent use of 5-fraction regimens in our cohort reflects institutional practice and may be associated with a lower risk of vertebral compression fractures than regimens with higher doses per fraction; notably, the single fracture in our cohort occurred after 24 Gy in 2 fractions, although our data do not allow a comparison between regimens. In addition, 40 Gy in 5 fractions, the most frequently prescribed regimen in our cohort, was also the predominant SBRT regimen in the prospective phase II trial by So et al. [27].

Several limitations must be acknowledged. First, the retrospective design introduces the risk of selection bias, as patients referred for SBRT may inherently represent a subgroup with more favorable performance status or limited disease burden. The indication for SBRT and the selection of lesions were decided by the local multidisciplinary tumor boards, and treatment intent was not recorded as a separate variable. Second, the sample size remains modest despite the multicenter nature of the study, which restricts the statistical power for subgroup analyses. Accordingly, all prognostic analyses should be considered exploratory and hypothesis-generating. Third, multiple lesions within the same patient are not statistically independent, which we addressed by patient-level bootstrapping. Fourth, the absence of a comparative control group prevents direct assessment of the incremental benefit of SBRT over systemic therapy alone; given patient selection and the further systemic therapies most patients received after SBRT, OS and PFS cannot be attributed to SBRT. Fifth, no data on pain response and quality of life were collected, which is particularly relevant for bone metastases, and retrospective assessment may underestimate low-grade and late adverse events. Sixth, local control of bone metastases after SBRT was not assessed by central imaging review. Finally, the study period spans more than a decade, during which diagnostic imaging, radiation techniques, and systemic therapies evolved substantially, potentially introducing temporal confounding. In addition, the cohort was heterogeneous regarding primary tumor site, metastatic burden, systemic therapy, and fractionation.

## 5 Conclusion

In this multicenter cohort, SBRT for HNSCC-derived bone metastases was associated with high local control and a low incidence of severe adverse events and post-treatment fractures. Despite limited PFS, durable OS was observed in a subset of carefully selected patients. Prospective randomized studies are warranted to define the role of SBRT for HNSCC-derived bone metastases and to identify the patients most likely to benefit from this approach, both regarding oncological efficacy and analgesic effects.

## CRediT authorship contribution statement

**Alexander Rühle:** Writing – original draft, Supervision, Project administration, Conceptualization. **Sebastian Schäfer:** Writing – original draft, Visualization, Software, Formal analysis, Data curation. **Isabell Prager:** Writing – review & editing, Investigation, Conceptualization. **Mauro Loi:** Writing – review & editing, Investigation. **Pierluigi Bonomo:** Writing – review & editing, Investigation. **Viola Salvestrini:** Writing – review & editing, Investigation. **Marek Slavik:** Writing – review & editing, Investigation. **Petr Burkon:** Writing – review & editing, Investigation. **Fabian Lohaus:** Writing – review & editing, Investigation. **Kenneth Klischies:** Writing – review & editing, Investigation. **Olaf Wittenstein:** Writing – review & editing, Investigation. **Anna Sabrina Schunn:** Writing – review & editing, Investigation. **Sophia Drabke:** Writing – review & editing, Investigation. **Thomas Weissmann:** Writing – review & editing, Investigation. **Jochen Willner:** Writing – review & editing, Investigation. **Camilla von Wachter:** Writing – review & editing, Investigation. **Esmée L. Looman:** Writing – review & editing, Investigation. **Panagiotis Balermpas:** Writing – review & editing, Investigation. **Matthias Guckenberger:** Writing – review & editing. **Sebastian Michael Ritter:** Writing – review & editing, Investigation. **Christos Moustakis:** Writing – review & editing, Conceptualization. **Ahmed Allam Mohamed:** Writing – review & editing, Investigation. **Thomas B. Brunner:** Writing – review & editing, Investigation. **Oliver Blanck:** Writing – review & editing, Investigation. **Judit Boda-Heggemann:** Writing – review & editing, Investigation. **Nils H. Nicolay:** Writing – review & editing, Supervision, Resources, Project administration, Conceptualization. **Franziska Nägler:** Writing – review & editing, Writing – original draft, Project administration, Methodology, Investigation, Data curation, Conceptualization.

## Ethics declaration

Informed consent and patient details: Written informed consent was obtained where required by local regulations; for the remaining patients, the requirement for informed consent was waived by the responsible ethics committees owing to the retrospective nature of the study. All data were pseudonymized, and the privacy rights of participants have been observed.

Studies in Human: This study was performed in compliance with relevant laws, regulatory frameworks and guidelines where the research took place. This study was approved by the Ethics Advisory Board at Leipzig University (Approval No. 127/24-ek).

## Funding

This research received funding from the “Roland Ernst Stiftung für Gesundheitswesen”. The funding body had no role in the design of the study, data collection and analysis, decision to publish, or preparation of the manuscript.

## Declaration of competing interest

The authors declare that they have no known competing financial interests or personal relationships that could have appeared to influence the work reported in this paper.

## References

[1] Kristensen M.H., Holm A.I.S., Hansen C.R., Zukauskaite R., Samsøe E., Maare C. (2024). High-dose loco-regional pattern of failure after primary radiotherapy in p16 positive and negative head and neck squamous cell carcinoma - a DAHANCA 19 study. Clin Transl Radiat Oncol.

[2] Chelvarajah R.C., Liu Y., Su J., Abdalaty A.H., Bratman S., John Cho B.C. (2025). Factors associated with detection of oligometastatic recurrence and outcome following definitive (chemo)radiotherapy for oropharyngeal carcinoma. Oral Oncol.

[3] Kjems J., Elisabet Håkansson K., Andrup Kristensen C., Grau Eriksen J., Horsholt Kristensen M., Ivalu Sander Holm A. (2023). The influence of tumor volume on the risk of distant metastases in head and neck squamous cell carcinomas. Radiother Oncol.

[4] Kjems J., Laursen M.R.T., Kristensen C.A., Gothelf A.B., Bernsdorf M., Specht L. (2023). The potential for local ablative therapy of oligometastases in head and neck squamous cell carcinoma: a real-world data analysis. Acta Oncol.

[5] Christ S.M., Pohl K., Muehlematter U.J., Heesen P., Kühnis A., Willmann J. (2022). Imaging-based prevalence of oligometastatic disease: a single-center cross-sectional study. Int J Radiat Oncol Biol Phys.

[6] Palma D.A., Olson R., Harrow S., Gaede S., Louie A.V., Haasbeek C. (2020). Stereotactic ablative radiotherapy for the comprehensive treatment of oligometastatic cancers: long-term results of the SABR-COMET phase II randomized trial. J Clin Oncol.

[7] Nägler F., Vorbach S., Mohamed A.A., Thaqi S., Adebahr S., Ehret F. (2025). Pulmonary stereotactic body radiotherapy of oligometastatic head-and-neck squamous cell carcinoma - a multicenter retrospective study. Int J Radiat Oncol Biol Phys.

[8] Thariat J., Bosset M., Falcoz A., Vernerey D., Pointreau Y., Racadot S. (2025). Survival without quality of life deterioration in the GORTEC 2014-04 "OMET" randomised phase 2 trial in head and neck cancer patients with oligometastases using stereotactic ablative radiotherapy (SABR)-alone or chemotherapy SABR. Int J Radiat Oncol Biol Phys.

[9] Mutsaers A., Akingbade A., Louie A.V., Id Said B., Zhang L., Poon I. (2024). Stereotactic body radiotherapy for extracranial oligometastatic disease from head and neck primary cancers: a systematic review and meta-analysis. Cancers (Basel).

[10] Lehyanti J., Even C., Fessart E., Wagner-Ballon C., Moreira A., Houessinon A. (2024). Management of oligometastatic head and neck squamous cell carcinoma: a systematic review. Oral Oncol.

[11] Bostel T., Rühle A., Rackwitz T., Mayer A., Klodt T., Oebel L. (2020). The role of palliative radiotherapy in the treatment of spinal bone metastases from head and neck tumors-a multicenter analysis of a rare event. Cancers (Basel).

[12] Hasegawa T., Tanakura M., Takeda D., Sakakibara A., Akashi M., Minamikawa T. (2015). Risk factors associated with distant metastasis in patients with oral squamous cell carcinoma. Otolaryngol Head Neck Surg.

[13] Grisanti S., Bianchi S., Locati L.D., Triggiani L., Vecchio S., Bonetta A. (2019). Bone metastases from head and neck malignancies: prognostic factors and skeletal-related events. PLoS One.

[14] Swain M., Ghosh-Laskar S., McDowell L., Henson C., Bonomo P., Kannan S. (2025). Patterns of care in de-novo oligo-metastatic and oligo-recurrent head and neck cancers: a Head and Neck Cancer International Group (HNCIG) survey. Oral Oncol.

[15] Zhu Y., Xue F. (2025). Clinical outcomes and prognostic factors of stereotactic body radiation therapy in metastatic nasopharyngeal carcinoma. Ther Adv Med Oncol.

[16] Yang J., Liao W., Su S., Zeng N., Zhang S., He J. (2024). Long-term outcomes of metastasis-directed stereotactic body radiation therapy in metastatic nasopharyngeal carcinoma. Cancer Med.

[17] Franzese C., Badalamenti M., Teriaca A., De Virgilio A., Mercante G., Cavina R. (2021). Metastasis-directed stereotactic body radiation therapy in the management of oligometastatic head and neck cancer. J Cancer Res Clin Oncol.

[18] Alzibdeh A., Wahbeh L., Taha S.A., Qambar M., Mousa A.A., Khader J. (2025). Metastasis-directed stereotactic body radiation therapy in oligometastatic and oligoprogressive solid malignancy: outcomes and effect on systemic treatment. JCO Glob Oncol.

[19] Guckenberger M., Lievens Y., Bouma A.B., Collette L., Dekker A., deSouza N.M. (2020). Characterisation and classification of oligometastatic disease: a European Society for Radiotherapy and Oncology and European Organisation for Research and Treatment of Cancer consensus recommendation. Lancet Oncol.

[20] Wilke L., Andratschke N., Blanck O., Brunner T.B., Combs S.E., Grosu A.L. (2019). ICRU report 91 on prescribing, recording, and reporting of stereotactic treatments with small photon beams : statement from the DEGRO/DGMP working group stereotactic radiotherapy and radiosurgery. Strahlenther Onkol.

[21] Brunner T.B., Boda-Heggemann J., Bürgy D., Corradini S., Dieckmann U.K., Gawish A. (2024). Dose prescription for stereotactic body radiotherapy: general and organ-specific consensus statement from the DEGRO/DGMP working group stereotactic radiotherapy and radiosurgery. Strahlenther Onkol.

[22] Singh R., Valluri A., Lehrer E.J., Cao Y., Upadhyay R., Trifiletti D.M. (2024). Clinical outcomes after stereotactic body radiation therapy for nonspinal bone metastases: a systematic review and meta-analysis. Int J Radiat Oncol Biol Phys.

[23] Guninski R.S., Cuccia F., Alongi F., Andratschke N., Belka C., Bellut D. (2024). Efficacy and safety of SBRT for spine metastases: a systematic review and meta-analysis for preparation of an ESTRO practice guideline. Radiother Oncol.

[24] Xu P., Liu D., Zhou J., Tang Z., Wang S., Huang Y. (2025). Survival analysis of patients with metastatic head and neck squamous cell carcinoma treated with metastasis-directed radiotherapy and immunotherapy. Radiat Oncol.

[25] Zhang X., Yan J., Lei Q., Neo J., Tan S.H., Shu X. (2025). A randomized, multicenter, phase 2 trial of camrelizumab with or without metastasis-directed stereotactic body radiation therapy in recurrent or metastatic nasopharyngeal carcinoma. Int J Radiat Oncol Biol Phys.

[26] Guenne C., Fayette J., Cosmidis A., Fuchsmann C., Tartas S., Favrel V. (2014). Curative treatment can be an option for patients with metastatic squamous cell cancer of the head and neck. Drug Des Devel Ther.

[27] So J., Droznin A.D., Tiwari P., Chen Y.H., Chau N.G., Margalit D.N. (2026). Stereotactic body radiation therapy with pembrolizumab in programmed cell death protein 1 inhibitor-refractory recurrent or metastatic head and neck squamous cell carcinoma: a phase 2 trial. Int J Radiat Oncol Biol Phys.

[28] Mercier C., Billiet C., Ost P., Vandecasteele K., De Kerf G., Claessens M. (2025). Dose-escalated stereotactic versus conventional radiotherapy for painful bone metastases (ROBOMET): a multicenter, patient-blinded randomized clinical trial. J Clin Oncol.

[29] Wu J.S., Wong R., Johnston M., Bezjak A., Whelan T. (2003). Meta-analysis of dose-fractionation radiotherapy trials for the palliation of painful bone metastases. Int J Radiat Oncol Biol Phys.

[30] Sahgal A., Myrehaug S.D., Siva S., Masucci G.L., Maralani P.J., Brundage M. (2021). Stereotactic body radiotherapy versus conventional external beam radiotherapy in patients with painful spinal metastases: an open-label, multicentre, randomised, controlled, phase 2/3 trial. Lancet Oncol.

